# The Effect of Hydroxyapatite Nanocrystals on Osseointegration of Titanium Implants: An *In Vivo* Rabbit Study

**DOI:** 10.1155/2014/171305

**Published:** 2014-01-19

**Authors:** Karin Breding, Ryo Jimbo, Mariko Hayashi, Ying Xue, Kamal Mustafa, Martin Andersson

**Affiliations:** ^1^Department of Chemical and Biological Engineering, Applied Surface Chemistry, Chalmers University of Technology, 41296 Gothenburg, Sweden; ^2^Department of Prosthodontics, Faculty of Odontology, Malmö University, 21421 Malmö, Sweden; ^3^Department of Clinical Dentistry, Center for Clinical Dental Research, University of Bergen, 5020 Bergen, Norway

## Abstract

Osseointegration is dependent on implant surface characteristics, including surface chemistry and topography. The presence of nanosized calcium phosphates on the implant surface is interesting to investigate since they affect both the nanotopography and surface chemistry, forming a bone mineral resembling surface. In this work, the osseointegration of titanium implants with and without the presence of hydroxyapatite (HA) nanocrystals has been evaluated *in vivo*. The integration was examined using removal torque measurements and real-time polymerase chain reaction (RT-PCR) analysis. The study was performed using two healing time points, 3 and 12 weeks. The results showed that the torque needed to remove the implants was insignificant between the non- and HA-coated implants, both at weeks 3 and 12. The RT-PCR, however, showed significant differences for osteoblast, osteoclast, and proinflammation markers when HA nanocrystals were present.

## 1. Introduction

During the last decades, novel implants have been developed with the aim of finding optimal conditions for osseointegration. It is well known that factors, such as surface chemistry and surface topography, influence osseointegration [1]. However, the biological influence of nanorough surfaces is a relatively new area of research and highly interesting since several studies have indicated that nanotopography can enhance osseointegration [2–4]. When combining the two surface entities, nanoroughness and bone-like chemistry, for example, by using nanosized hydroxyapatite (HA), a synergistic effect can be generated [2]. HA is a widely known and frequently used material, which is often being utilized as relatively thick implant coatings. Recently, techniques have been developed which made it possible to coat implants with a monolayer of HA nanoparticles. Such thin HA layers have proven to increase the wettability and thereby increase the surface energy of the implant. Increasing the surface energy is speculated to affect the cell and protein adhesion resulting in improved osseointegration [5, 6]. Moreover, the adsorption of plasma proteins, such as fibronectin and laminin, has been suggested to increase when implants have been surface-modified with calcium and phosphorous [7].

There are many suggested techniques to determine if osseointegration has occurred [8–13]. Commonly, the removal torque of the implant and histology of the bone-implant interface are examined, both after specific healing times. Most often these two methods are combined, even though not on the same implant, in order to reach a more complete overview of the integration. However, contradictory results have been observed between studies when these two evaluation techniques have been used. HA nanoparticles have been shown to improve the bone-to-implant contact (BIC) when deposited onto electropolished cylindrical-shaped titanium implants [14]. Also, the removal torque has been measured to increase when HA nanoparticles are present on screw-shaped titanium implants [15]. However, Svanborg et al. failed to show any significant differences when HA nano coated titanium screws were compared to uncoated ones [16]. Discrepancies, such as these, can have many explanations, including differences in implant design, surgical techniques, and evaluation methodology. Recently, novel techniques have emerged and are utilized to evaluate osseointegration, some of which have been suggested to more accurately determine the outcome of nanostructured implants. Studies utilizing gene expression techniques and nanoindentation have shown that the effect of the nanostructured HA coating significantly enhanced the mineralization properties [17, 18]. This was evident even when the histological or biomechanical evaluation approaches did not present significant differences, indicating that the conventional evaluation approaches may be too coarse to investigate the effect of the nanostructure. None of the aforementioned methods is yet the ultimate single method to measure osseointegration, especially when it comes to the influence of nanostructures.

In the present work, we have studied the osseointegration of sand-blasted and acid-etched titanium screw-shaped implants with and without nanosized HA coating. The integration was evaluated using a rabbit study, which was performed at three and twelve weeks. The integration was examined using removal torque measurements and real-time polymerase chain reaction (RT-PCR) analysis.

## 2. Materials and Methods

### 2.1. Implant Surface Preparation

Twenty threaded implants measuring 6 mm in length and 3.3 mm in diameter were produced out of Ti6A4V. All implants were sand blasted and acid etched according to a procedure used in a previous study [17]. Half of them were coated with nanosized hydroxyapatite (HA), and the other half was left untreated and used as control. 50 *μ*L of a HA-particle coating dispersion was poured onto each implant followed by spinning the implant combined with applying a flow of pressurized nitrogen gas, a procedure resulting in a thin layer of particles deposited onto the implant surface. The implants were thereafter heat treated at 450°C for 5 min in an oxygen atmosphere. Four circular discs were also produced and sand blasted and acid etched using the same protocol as for the implants. Two of the discs were also coated with the nanosized HA particles as described above.

### 2.2. Scanning Electron Microscopy (SEM)

A scanning electron microscope (LEO Ultra FEG 55, Zeiss, Oberkochen, Germany) was used to investigate the surface morphology of the implants and the circular discs. The analysis was performed at an accelerating voltage of 5 kV using secondary electron detectors. Two implants were randomly selected from each group. Each implant was analyzed at nine positions (thread top, thread valley, and flanks × 3). The four circular discs were all analyzed at two randomly selected areas.

### 2.3. X-Ray Photoelectron Spectroscopy (XPS)

The chemical composition of the outermost surface of the implants was analyzed using XPS (PHI 5500 XPS, PerkinElmer, Waltham, MA, USA). XPS survey spectra were obtained using a *α* excitation source operating at 250 W with an angle of 45°. Two implants were randomly selected from each group. Each implant was analyzed at two positions.

### 2.4. Atomic Force Microscopy (AFM)

The surface roughness on the nanometer scale was analyzed using an AFM (INTEGRA Probe NanoLaboratory, NT-MDT, Zelenograd, Russia). Analyses were performed on the circular discs (two noncoated and two HA-coated). Each disc was scanned at three randomly selected areas and was recorded in two different fields of view, that is, 5 × 5 *μ*m and 1 × 1 *μ*m. The microscope was set to operate in tapping mode and silicon probes (Tap300Al-G, Budget Sensors, resonance freq. 300 kHz) were used. Analysis was performed using the software NOVA 1.0.26 RC1 (NTEGRA Probe NanoLaboratory, NT-MDT, Zelenograd, Russia). Errors of bow and tilt were corrected with a third order subtraction before average height deviation (*S*_*a*_) was calculated.

### 2.5. Animals, Implantation, and Sample Preparation

Twenty adult Swedish lop-eared rabbits (mean weight 4.2 kg) were used. Two implants (one HA-coated and one noncoated used as a reference) were inserted into the proximal part of the left and right tibiae, respectively. Before surgery, the surgical site was shaved and disinfected with 70% ethanol and 70% chlorhexidine. The animals were anesthetized with intramuscular injections of a mixture of 0.15 mL/kg medetomidine (1 mg/mL Dormitor; Orion Pharma, Sollentuna, Sweden) and 0.35 mL/kg ketamine hydrochloride (50 mg/mL Ketlar; Pfizer AB, Sollentuna, Sweden). Lidocaine hydrochloride (Xylocaine; AstraZeneca AB, Södertälje, Sweden) was administered as the local anesthetic at each insertion site at a dose of 1 mL. Osteotomy was prepared with a series of drills and was finalized at a diameter of 2.9 mm, and the implants were thereafter inserted. Postoperatively, buprenorphine hydrochloride (0.5 mL Temgesic; Reckitt Benckiser, Slough, UK) was given as an analgesic for 3 days.

### 2.6. Removal Torque

The rabbits were sacrificed by an anesthetic overdose at weeks 3 and 12 after surgery and the implants and surrounding tissues were removed en bloc. Subsequently, the removal torque needed to unscrew the implant was measured using an electrically controlled removal torque unit [19]. After the removal torque measurements, implants and surrounding bone tissue were placed in RNAlater solution and frozen at −80°C to preserve the mRNA for RT-PCR analysis.

### 2.7. RNA Extraction and Real-Time Reverse-Transcription PCR

RNA extraction from the bone tissue was performed using QiaZol solution (Qiagen GmbH) combined with RNA Tissue Kit SII. To reduce DNA contamination during extraction, all samples were DNase-treated with RNase-free DNase (Qiagen GmbH). RNA quantification was performed using a NanoDrop Spectrophotometer (ThermoScientific NanoDrop Technologies, Wilmington, DE, USA).

The amounts of RNA in the samples were normalized to 50 ng/*μ*L and reverse-transcribed in single 50 *μ*L reactions (25 *μ*L RT Mix and 25 *μ*L sample). All reverse transcriptions were performed using a high capacity cDNA reverse transcription kit (Applied Biosystems) to generate cDNA for relative quantification on mRNA. The cDNA samples were stored in −20°C until real-time PCR.

Real-time quantitative reverse-transcription PCR (RT-PCR) was performed in 20 *μ*L reaction in triplicate for each sample, with custom-designed primers (Table 1) of SYBR green detection (PrimerDesign Ltd, Southampton, UK). Each PCR reaction contained 1 *μ*L Primer, 10 *μ*L Master Mix, 4 *μ*L water, and 5 *μ*L cDNA template and was performed using a a 96-well StepOnePlus system (Applied Biosystems, Foster City, CA, USA). StepOne Software v2.3 was used for analysis and the data was normalized by a comparative Ct or ΔΔCt method to get the relative mRNA expression [20, 21]. The control group was set as reference and normalized with the test group in the calculations. *β*-Actin was used as endogenous control to normalize the input difference of the samples. Both osteogenic markers; ALP, ATPase, Calcitonin receptor, Collagen I, IGF-1, Osteocalcin, Runx2 and TRAP, and Inflammation markers; IL-6, IL-10 and TNF-*α* were analyzed, Table 1.

### 2.8. Statistical Analysis

The statistical analysis for the removal torque was performed using SAS proc glm and proc mixed (SAS Institute Inc, USA). The analysis was performed using three-way analysis of variance. The rabbit was regarded as random; time (three and twelve weeks) and treatment (reference and HA-coated) were regarded as fixed factors. The significance level was set at 0.05.

## 3. Results

### 3.1. Material Characterization

#### 3.1.1. Scanning Electron Microscopy (SEM)

**Figure 1:**
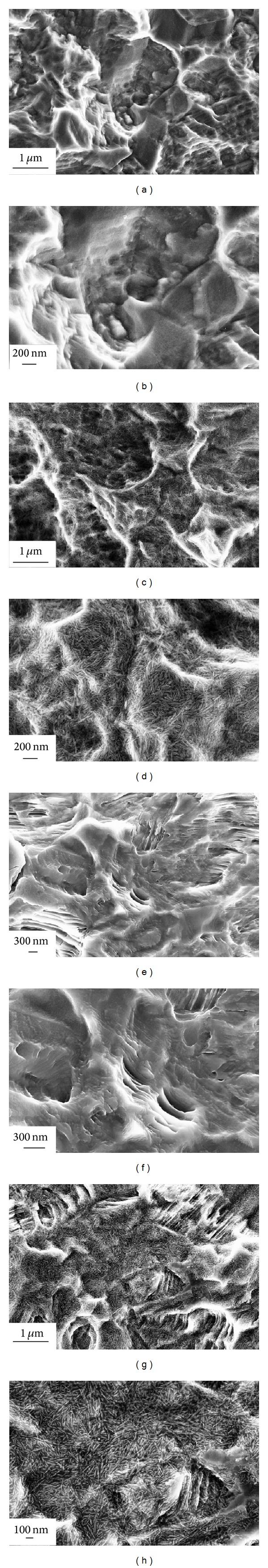
SEM micrographs of noncoated implant at magnifications 40 kX and 80 kX ((a)-(b)) and HA-coated implant at mag. 40 kX and 80 kX ((c)-(d)). SEM images of noncoated disc at mag. 40 kX and 80 kX ((e)-(f)) and HA-coated disc at mag. 40 kX and 80 kX ((g)-(h)).

In Figure 1, SEM images of both noncoated pure titanium and HA-coated titanium screw-shaped implants are shown. At the higher magnification (80 kX) the nanometer-sized HA crystals are clearly seen as elongated particles deposited onto the surface of the HA-coated implants. The particles follow the underlying topography forming an evenly distributed monolayer. In Figures 1(e)–1(h), SEM micrographs of the noncoated and the HA-coated titanium discs are shown. In these images it is seen that the surface morphology of the discs differs somewhat from the surface morphology of the implants. However, the HA crystal layers of the coated discs look similar to the layer onto the coated implants.

#### 3.1.2. X-Ray Photoelectron Spectroscopy (XPS)

**Figure 2:**
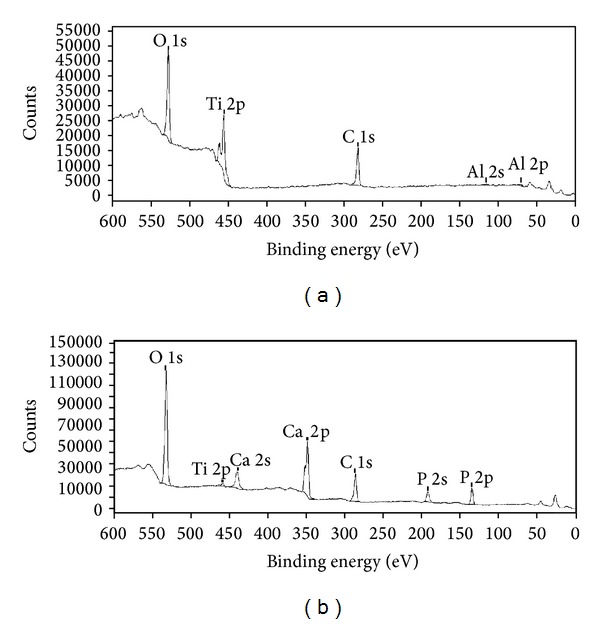
XPS-spectra for (a) noncoated implant and (b) HA-coated implant. The binding energy was monitored between 0 and 600 eV.

XPS survey spectra for noncoated and HA-coated implants are presented in Figure 2. The XPS-spectrum for the HA-coated implants revealed that the surface contained calcium and phosphorous, which were not observed on the noncoated surface. The amount of carbon on the noncoated and the HA-coated implants was similar.

#### 3.1.3. Atomic Force Microscopy (AFM)

**Figure 3:**
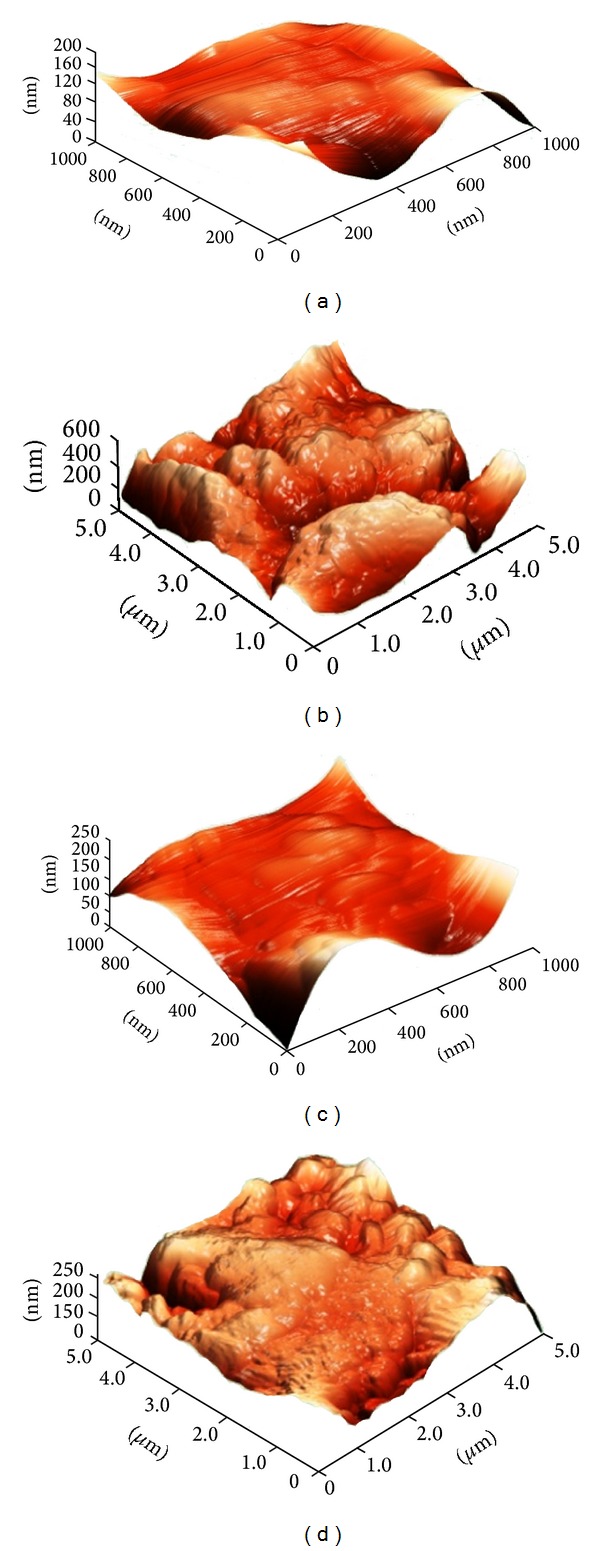
3D AFM images of a noncoated disc ((a) and (b)) and a HA-coated disc ((c) and (d)). Measurements were performed at two different fields of view, 1 *μ*m × 1 *μ*m ((a) and (c)) and 5 *μ*m × 5 *μ*m ((b) and (d)).

In Figure 3, AFM micrographs obtained in height mode for both the noncoated and the HA-coated discs are presented. From the topographical images, no visual differences could be seen between the two surface types. The average values from the surface roughness analysis are shown in Table 2. The HA-coated discs showed notably lower *S*_*a*_ values for both fields of view (5 *μ*m × 5 *μ*m and 1 *μ*m × 1 *μ*m) compared to the noncoated discs.

### 3.2. *In Vivo*

#### 3.2.1. Removal Torque

The results from the removal torque tests after 3 and 12 weeks of healing are presented in Table 3. The 12-week removal torque measurements were performed on 9 animals; due to that one rabbit died during insertion surgery. Statistical analysis showed that no significant difference could be detected for both weeks 3 and 12 (*P* = 0.19, resp., 0.06). Comparison between the two healing times was performed using three-way analysis of variance. The animals were regarded as random and time (week three/week twelve) and treatment (noncoated/HA-coated) were regarded as fixed factors. Analysis by SAS proc glm and proc mixed. The three-way analysis showed no significant differences between the noncoated and the HA-coated implants (*P* = 0.28, DF = 17, *t*-value −1.12, *α* 0.05).

#### 3.2.2. Real-Time Polymerase Chain Reaction (RT-PCR)

**Figure 4:**
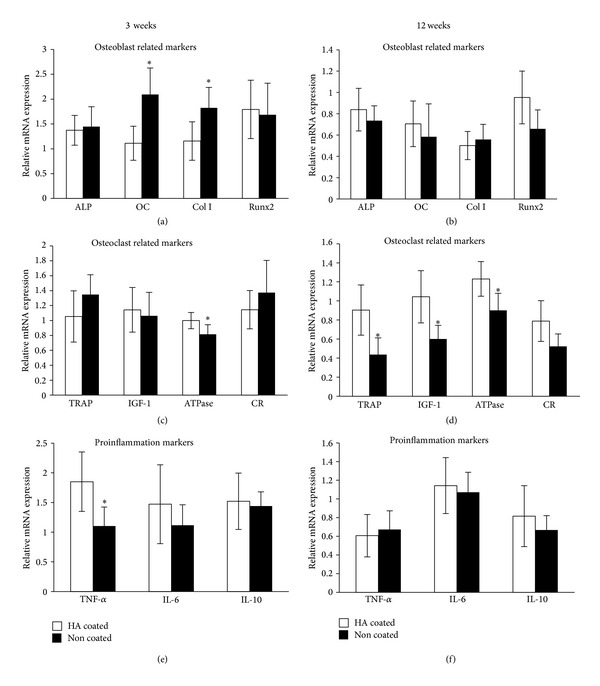
Results from RT-PCR analysis of selected genes (ALP, osteocalcin, Collagen I, Runx2, TRAP, IGF-1, ATPase, Calcitonin receptor, TNF-*α*, IL-6, and IL-10) after three weeks and twelve weeks, respectively. The genes were normalized with the housekeeping gene *β*-actin, *P < 0.05. Please note that the genes cannot be compared to each other, only between experimental groups, since the genes are normalized to actin.

Results from the osteoblast, osteoclast, and proinflammation markers in the RT-PCR analysis are presented in Figures 4(a)–4(f). At three weeks of healing, gene expression of osteocalcin and Collagen I was significantly higher for the HA-coated implants compared to the noncoated implants (*P* = 0.046, resp., *P* = 0.042), whereas the gene expression for ATPase and TNF-*α* was significantly lower for the HA-coated implant compared to the noncoated implants (*P* = 0.008, resp., *P* = 0.0231). At 12 weeks of healing the gene expression of TRAP, IGF-1, and ATPase was significantly lower (*P* = 0.01; *P* = 0.02, resp., *P* = 0.007) for the HA-coated implants compared to the noncoated implants. For the other markers no significant differences were detected.

## 4. Discussion

In this study, we evaluated the osseointegration properties of titanium implants coated with HA nanoparticles and compared them with noncoated counterparts. The implant surfaces were characterized and it was shown that both nanotopography and surface chemistry were different between the two investigated implants. For the HA-coated implants, SEM showed that elongated particles were present on the surfaces, which most likely are HA crystals. In order to evaluate the nanotopography of the surfaces using AFM, titanium discs were prepared using the same procedure as for the implant screws. SEM revealed, however, that the surface morphology of the discs differed from the implants. Despite these differences, the HA particles seemed to be deposited in a similar fashion on the discs as on the implants. As a consequence, the differences in measured topography on the discs, which is attributed to the HA nanoparticles, are believed to be directly comparable to the ones on the implants. From the AFM results, it was observed that the surface roughness was lower when HA particles were applied onto the surface, which indicates that the particles smoothened out smaller surface features.

The removal torque was measured after 3 and 12 weeks of healing in the rabbit tibia. Statistical calculations showed no significance between the two surfaces at neither of the two time periods. Not even when all HA-coated implants were compared to the noncoated ones could significance be detected. In a previous study, which was performed using the same types of implants, it was shown that the removal torque was significantly higher for the HA-coated surface compared to a noncoated surface only after 2 weeks of healing. In the same study, no significant difference was observed after 4 weeks of healing [18]. This is an indication that the effect of the nano-HA is especially significant at earlier healing periods and biomechanically the values seem to present no differences after bone maturation. This has been a general tendency with nanosized HA deposited onto titanium surfaces [2, 16, 20, 21]. Furthermore, removal torque is indeed a course evaluation technique, since the values may be influenced by different factors, such as macrogeometry or microtopography. It has been suggested that in order to detect the differences generated by nanotopography, other biological evaluation techniques, such as the modified pull out testing, may be suitable [22–24]. However, the use of a screw type model in animal studies is of great value, since this may provide valuable information for the actual clinical implant performance. Thus, in order to detect the detailed differences generated at the nanolevel, different evaluation techniques have been utilized and have provided some interesting results. For example, nanomechanical testing to evaluate the bone nanomechanical properties has shown that indeed there are differences even when no differences were detected with the conventional methods [17]. Furthermore, other state of the art techniques such as the use of micro-CT have provided the possibility to further investigate the unique bone-forming properties to both micro- and nanotopography [25–27]. Sarve et al. have further explored the possibility of obtaining improved boneimplant interfacial images with the use of s*μ*CT and have shown that the technique can evaluate the bone-healing properties to surfaces possessing nanotopography [28].

Another detailed evaluation that can possibly explore the genetic mechanisms of the responses to the nanostructured surface is the gene expression. After 3 weeks, significantly higher expressions of osteoblast marking genes, osteocalcin, and Collagen I were detected in the tissue surrounding the HA-coated implants. This indicates a higher osteoprogenitor activity for the modified surface [29]. This increase in the expression of osteogenic genes was not observed after 12 weeks. Interestingly, the osteoclast related marker, TNF-*α*, was significantly downregulated in the surrounding tissue of the HA-coated implants after 3 weeks. Reportedly, TNF-*α* contributes to bone loss by inhibiting the IGF-I and ALP genes [30, 31]. The expression of the transmembrane ATPase was lowered around the surrounding tissue of both the 3- and 12-week HA-coated implants. It has been known that a lowered number of ATPase could affect the cell metabolism [32], and this may have influenced the bone turnover rate of the nanostructured surface. At 12 weeks both the TRAP and the IGF-I were significantly lowered for the tissue surrounding the HA implants. Within the limitation of the current study, it is difficult to fully interpret the obtained results since the RT-PCR is still a phenomenological investigation where the researcher selects the gene of interest; however, it is evident that the differences in surface nanotopography and chemistry obviously influenced the gene expression, which was not detectable by the removal torque. In order to further investigate the detailed genetic mechanism, methodologies to detect the signaling pathway may be useful.

## 5. Conclusions

In this work, the osseointegration of screw-shaped titanium implants coated with hydroxyapatite (HA) nanocrystals was evaluated *in vivo*. The results demonstrated that the torque needed to remove the implants was insignificant between pure titanium and HA-coated implants, both at weeks 3 and 12 of healing. RT-PCR performed on osteoblast, osteoclast, and proinflammation markers, however, showed significant differences when HA nanocrystals were present. The results show that nanosized HA crystals deposited onto implants do have a biological effect; however, it is not always detectable using removal torque measurements.

## Figures and Tables

**Table 1:** The gene expressions that were analyzed.

Gene	Primer sequence	Tm	Amplicon size (bp)	Primer source
ALP	S TGGACCTCGTGGACATCTG	75	80	*Oryctolagus cuniculus *
	A CAGGAGTTCAGTGCGGTTC			
ATPase	S CCTGGCTATTGGCTGTTACG	77.7	98	*Oryctolagus cuniculus *
	A GCTGGTAGAAGGACACTCTTG			
Calcitonin receptor	S CGTTCACTCCTGAAAACTACA	72.6	128	*Oryctolagus cuniculus *
	A GCAACCAAGACTAATGAAACA			
Collagen I	S GGAAACGATGGTGCTACTGG	80.4	83	*Oryctolagus cuniculus *
	A CCGACAGCTCCAGGGAAG			
IGF-1	S CCGACATGCCCAAGACTCA	70.3	81	*Oryctolagus cuniculus *
	A TACTTCCTTTCCTTCTCCTCTGA			
IL-6	S GAGGAAAGAGATGTGTGACCAT	73.5	104	*Oryctolagus cuniculus *
	A AGCATCCGTCTTCTTCTATCAG			
IL-10	S CCGACTGAGGCTTCCATTCC	73.3	75	*Oryctolagus cuniculus *
	A CAGAGGGTAAGAGGGAGCT			
Osteocalcin	S GCTCAHCCTTCGTGTCCAAG	77.8	70	*Oryctolagus cuniculus *
	A CCGTCGATCAGTTGGCGC			
Runx2	S GCAGTTCCCAAGCATTTCATC	72.8	81	*Oryctolagus cuniculus *
	A GTGTAAGTAAAGGTGGCTGGATA			
TNF-*α*	S CTCACTACTCCCAGGTTCTCT	78.2	122	*Oryctolagus cuniculus *
	A TTGATGGCAGAGAGGAGGTT			
TRAP	S GCTACCTCCGCTTCCACTA	78.5	129	*Oryctolagus cuniculus *
	A GCAGCCTGGTCTTGAAGAG			
*β*-Actin	S CACCCTGATGCTCAAGTACC	76.4	96	*Oryctolagus cuniculus *
	A CGCAGCTCGTTGTAGAAGG			

**Table 2:** Average *S*_*a*_ ± SD values for HA coated and noncoated discs.

	HA coated	Noncoated
Average *S*_*a*_, 1 × 1 *μ*m	4.9 ± 2.0 nm	12.9 ± 5.6 nm
Average *S*_*a*_, 5 × 5 *μ*m	41.3 ± 11.8 nm	70.3 ± 13.3 nm
Number	6	7

**Table 3:** Mean removal torque values and statistical comparisons.

	Number	Mean (SD)·Ncm	Median (min; max)
Three weeks			
Noncoated	10	32.4 (8.1)	33.5 (16; 42)
HA coated	10	27.8 (9.1)	30.5 (10; 39)
Twelve weeks			
Noncoated	9	49.3 (11.1)	54 (32; 62)
HA coated	9	38.2 (13.4)	40 (21; 61)

Distribution of the removal torque differences between three weeks and twelve weeks.			
Rabbit	Difference (noncoated-HA coated)	Rabbit	Difference (noncoated-HA coated)

1	19	11	1
2	8	12	−3
3	7	13	2
4	13	14	−7
5	−15	15	30
6	3	16	8
7	−4	17	22
8	3	18	11
9	−3	19	—
10	15	20	36
